# Calmodulin influences MAPK signaling by binding KSR1

**DOI:** 10.1016/j.jbc.2021.100577

**Published:** 2021-03-23

**Authors:** Swetha Parvathaneni, Zhigang Li, David B. Sacks

**Affiliations:** Department of Laboratory Medicine, National Institutes of Health, Bethesda, Maryland, USA

**Keywords:** calmodulin, kinase suppressor of Ras1 (KSR1), mitogen-activated protein kinase (MAPK), calcium, protein–protein interaction, signaling, [Ca^2+^]_i_, intracellular free Ca^2+^ concentration, DMEM, Dulbecco’s modified Eagle’s medium, DMSO, dimethyl sulfoxide, EGF, epidermal growth factor, EGTA, ethylene glycol-bis(β-aminoethyl ether)-N,N,N′,N′-tetraacetic acid, ERK, extracellular signal-regulated kinase, FBS, fetal bovine serum, GST, glutathione *S*-transferase, IQGAP1, IQ motif–containing GTPase-activating protein, KSR1, kinase suppressor of Ras1, MAPK, mitogen-activated protein kinase, MEF, mouse embryonic fibroblast, NGF, nerve growth factor, pERK, phosphorylated ERK, PLA, proximity ligation assay, PVDF, polyvinylidene difluoride, SDS-PAGE, sodium dodecyl sulfate–polyacrylamide gel electrophoresis

## Abstract

The mitogen-activated protein kinase (MAPK) cascade is a fundamental signaling pathway that regulates cell fate decisions in response to external stimuli. Several scaffold proteins bind directly to kinase components of this pathway and regulate their activation by growth factors. One of the best studied MAPK scaffolds is kinase suppressor of Ras1 (KSR1), which is induced by epidermal growth factor (EGF) to translocate to the plasma membrane where it activates extracellular signal-regulated kinase (ERK). While Ca^2+^ has been shown to modulate MAPK signaling, the molecular mechanisms by which this occurs are incompletely understood. Here we tested the hypothesis that Ca^2+^ alters MAPK activity at least in part *via* KSR1. Using several approaches, including fusion proteins, immunoprecipitation, confocal microscopy, and a cell-permeable chemical inhibitor, we investigated the functional interaction between KSR1 and calmodulin. *In vitro* analysis with pure proteins reveals that calmodulin binds directly to KSR1. Moreover, endogenous calmodulin and KSR1 co-immunoprecipitate from mammalian cell lysates. Importantly, Ca^2+^ is required for the association between calmodulin and KSR1, both *in vitro* and in cells. The cell-permeable calmodulin antagonist CGS9343B significantly reduced activation of ERK by EGF in mouse embryo fibroblasts that overexpress KSR1, but not in control cells. Moreover, CGS9343B impaired the ability of EGF to induce KSR1 translocation to the plasma membrane and to stimulate formation of KSR1-ERK and KSR1-pERK (phosphorylated ERK) complexes in cells. Collectively, our data identify a previously unrecognized mechanism by which the scaffold protein KSR1 couples Ca^2+^ and calmodulin signaling to the MAPK cascade.

The mitogen-activated protein kinase (MAPK) pathway is a vital signal transduction cascade that regulates cell growth, cell proliferation, cell differentiation, apoptosis, and stress responses (1, 2). MAPKs are evolutionally conserved proteins that respond to diverse stimuli and link extracellular signals to fundamental cellular processes. The best studied of the MAPK pathways, the ERK module, comprises a linear cascade of the kinases Raf, MEK (MAP or ERK kinase), and ERK (extracellular signal-regulated kinase) (1, 2). The pathway can be activated by several stimuli, including growth factors, which bind to cell surface receptors. The growth factor receptors are activated by autophosphorylation, leading to stimulation of Ras, which activates Raf, thus coupling the receptors to the MAPK cascade (2). To facilitate optimal activation, the components of the ERK cascade are organized into complexes by scaffold proteins, which regulate their local concentration and subcellular localization (3, 4). In addition, scaffolds control the duration and magnitude of signaling (5). Scaffold proteins that modulate the ERK pathway include paxillin (6), MEK partner 1 (MP1) (7), IQ motif containing GTPase-activating protein 1 (IQGAP1) (8), and kinase suppressor of Ras1 (KSR1) (9).

KSR1 binds B-Raf, C-Raf, MEK, and ERK (10, 11, 12) to facilitate spatiotemporal activation of ERK signaling. In quiescent cells, KSR1 is constitutively associated with MEK and is located predominantly in the cytosol. Epidermal growth factor (EGF) induces translocation of a fraction of KSR1 to the plasma membrane, where it facilitates signal propagation and ERK activation by assembling Raf-MEK-ERK complexes (2). KSR1 is required for maximal activation of ERK (13). Several other proteins associate with KSR1 and influence its function. These include enzymes that regulate KSR1 phosphorylation, *e.g.*, protein phosphatase 2A (14) and casein kinase 2 (12), as well as 14-3-3 proteins (15), c-TAK1 (16), and IMP (impedes mitogenic signal propagation) (17).

Numerous extracellular regulators control cellular behavior *via* the second messenger molecule Ca^2+^ (18). Ca^2+^ signals are mediated through intracellular Ca^2+^-binding proteins, which couple the Ca^2+^ signal to cellular processes. Calmodulin is a highly conserved, ubiquitous protein that translates Ca^2+^ signals to cellular changes. On binding Ca^2+^, the conformation of calmodulin is altered, allowing it to bind to and influence the function of diverse proteins (19). Evidence from several investigators has demonstrated that Ca^2+^ and calmodulin impact MAPK signaling (20, 21). For example, synaptically evoked Ca^2+^ influx in some neurons activates ERK *via* cAMP (20). In other types of neurons, Ca^2+^/calmodulin activates Ras by binding to Ras guanine nucleotide-releasing factor (22). Calmodulin antagonists prevent ERK activation by nerve growth factor (NGF) or membrane depolarization in PC12 neuronal cells (21). By contrast, calmodulin inhibition increased ERK phosphorylation induced by EGF in Swiss 3T3 cells (23). While the combined evidence indicates that Ca^2+^ and calmodulin impact the MAPK pathway, the molecular mechanisms that underlie many of these findings are unknown.

In addition to the mechanisms outlined above, calmodulin has been shown to regulate MAPK *via* a scaffold protein. Prior work from both our laboratory and others documented that calmodulin modulates the interactions of IQGAP1 with several binding partners (24, 25, 26), including components of the MAPK pathway (27). In the presence of Ca^2+^, calmodulin inhibits the binding of IQGAP1 to B-Raf, which decreases the ability of EGF to activate B-Raf in fibroblasts (27). Here we tested the hypothesis that Ca^2+^/calmodulin also alters MAPK signaling *via* KSR1. We observe that calmodulin binds directly to KSR1 *in vitro* and co-immunoprecipitates with calmodulin from mammalian cells. Importantly, this interaction has functional significance. The specific calmodulin antagonist CGS9343B decreased the ability of EGF to induce ERK phosphorylation in cells that overexpress KSR1.

## Results

### KSR1 binds directly to calmodulin in Ca^2+^-regulated manner

**Figure 1:**
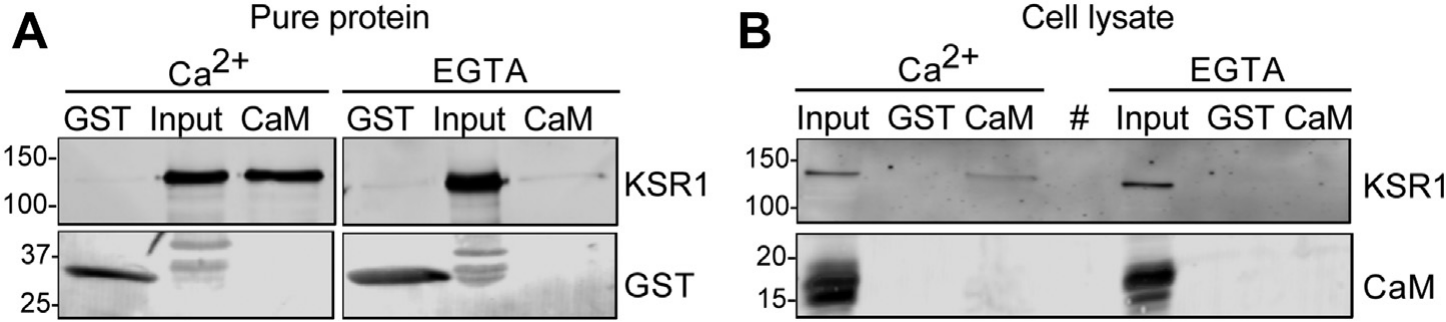
**KSR1 binds directly to calmodulin in a Ca**^**2+**^**-regulated manner.***A*, GST-Sepharose (GST) or calmodulin-Sepharose (CaM) was incubated with 500 ng of purified His-KSR1 in the presence of 1 mM Ca^2+^ or 1 mM EGTA. After washing the beads, attached proteins were resolved by SDS-PAGE and western blotting. Blots were probed with anti-KSR1 and anti-GST antibodies. Input is purified KSR1 not subjected to pull-down. The data are representative of two independent experiments. *B*, in total, 1 mg protein lysate prepared from KSR1+/+ MEFs in the presence of 1 mM Ca^2+^ or 1 mM EGTA was incubated with GST-Sepharose (GST) or calmodulin-Sepharose (CaM) beads. Samples were processed by SDS-PAGE and western blotting. Blots were probed with anti-KSR1 and anti-calmodulin (CaM) antibodies. Input is 20 μg lysate not subjected to pull-down. An empty lane is designated by (#). The data are representative of three independent experiments. The positions of migration of molecular mass markers are indicated on the *left*.

The possible interaction between calmodulin and KSR1 was examined by two approaches. To ascertain whether KSR1 binds directly to calmodulin, we used pure proteins. His-tagged full-length KSR1, purified from *Escherichia coli*, was incubated with calmodulin-Sepharose. Samples were resolved by SDS-PAGE, followed by western blotting. Analysis revealed that KSR1 binds to calmodulin in the presence of Ca^2+^ (Fig. 1*A*). Binding was specific as no KSR1 was detected in samples incubated with GST-Sepharose. Ca^2+^ regulates the interaction of calmodulin with numerous binding partners (19). Therefore, we repeated the analysis, but used buffer containing EGTA, which chelates Ca^2+^. No interaction of KSR1 with calmodulin is detected in the absence of Ca^2+^ (Fig. 1*A*). These data reveal direct binding between KSR1 and calmodulin that is dependent on Ca^2+^.

To determine whether calmodulin binds to KSR1 in a normal cell milieu, we incubated lysates from KSR1+/+ mouse embryo fibroblasts (MEFs) with calmodulin-Sepharose. Bound proteins were resolved by SDS-PAGE and western blotting. Probing blots with anti-KSR1 antibody revealed that KSR1 binds to calmodulin in the presence of Ca^2+^ (Fig. 1*B*). The absence of KSR1 from the negative control demonstrates binding specificity. As observed with pure protein, chelation of Ca^2+^ with EGTA abrogates the association between calmodulin and KSR1 in cell lysates (Fig. 1*B*). These data indicate that KSR1 in cell lysates binds to Ca^2+^/calmodulin.

### Calmodulin and KSR1 interact in cells

**Figure 2:**
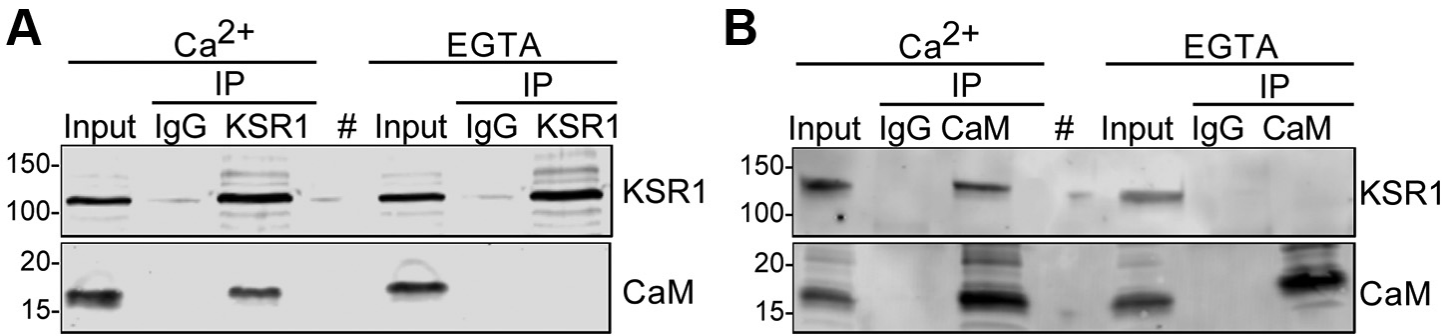
**Calmodulin and KSR1 interact in cells.***A*, KSR1+/+ MEFs were lysed in buffer containing 1 mM Ca^2+^ or 1 mM EGTA and 1 mg of cell lysate was immunoprecipitated (IP) with anti-KSR1 antibody. A sample precipitated with mouse IgG was used as the negative control. Lysates not subjected to immunoprecipitation (Input) were processed in parallel. Samples were resolved by western blotting and probed with anti-KSR1 and anti-calmodulin (CaM) antibodies. An empty lane is designated by (#). The data are representative of three independent experiments. *B*, in total, 1 mg protein lysate from KSR1+/+ MEFs was immunoprecipitated with anti-calmodulin (CaM) antibody in the presence of Ca^2+^ or EGTA. A sample precipitated with mouse IgG was used as the negative control. Samples were processed as described for panel *A*. An empty lane is designated by (#). The data are representative of three independent experiments. The positions of migration of molecular mass markers are indicated on the *left*.

To evaluate whether KSR1 and endogenous calmodulin interact in cells, we used immunoprecipitation. KSR1 was immunoprecipitated from cell lysates. Western blotting revealed that endogenous calmodulin co-immunoprecipitated with KSR1 in the presence of Ca^2+^ (Fig. 2*A*) Consistent with our calmodulin-Sepharose data, addition of EGTA abrogated binding of calmodulin to KSR1. The absence of calmodulin from the samples precipitated with IgG validates the specificity of the interaction (Fig. 2*A*). To confirm these observations, we performed the reverse immunoprecipitation. Calmodulin was immunoprecipitated with a specific anti-calmodulin monoclonal antibody (28) and blots were probed for KSR1. We observed that KSR1 co-immunoprecipitated with calmodulin exclusively in the presence of Ca^2+^; chelation of Ca^2+^ with EGTA abrogated the interaction (Fig. 2*B*). The amounts of calmodulin immunoprecipitated by the anti-calmodulin antibody were the same under both conditions and were not influenced by Ca^2+^ (Fig. 2*B*). These data demonstrate that KSR1 and calmodulin form a complex in cell lysates and Ca^2+^ is required for the interaction.

### Identification of the calmodulin-binding region on KSR1

**Figure 3:**
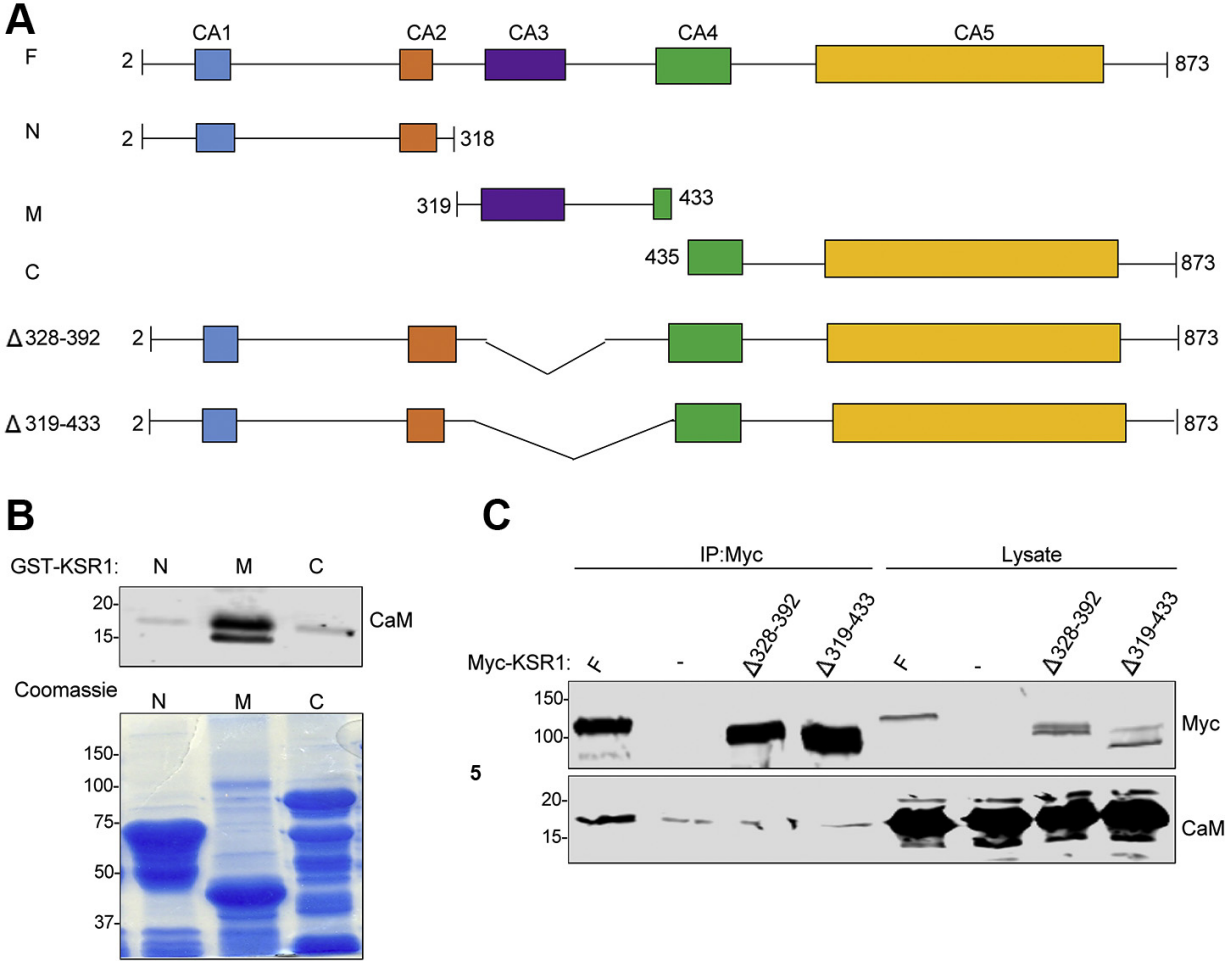
**Identification of the calmodulin-binding region in KSR1.***A*, schematic representation of KSR1 constructs. KSR1 contains five distinct domains, termed conserved area 1 (CA1) through CA5. The KSR1 constructs are full-length (F, amino acids 2–873), N (2–318), M (319–433), and C (435–873). KSR1Δ328–392 has amino acids 328–392 deleted and KSR1Δ319–433 has amino acids 319 to 433 deleted. *B*, GST-tagged fragments of KSR1-N, -M or -C were incubated with purified calmodulin. Complexes were pulled down with glutathione-Sepharose beads and analyzed by SDS-PAGE. The gel was cut at ∼25 kDa. The *lower portion* of the gel was processed by western blotting and probed with anti-calmodulin (CaM) antibody. The *upper portion* of the gel was stained with Coomassie *blue*. Data are representative of two independent experiments. *C*, HEK293 cells were transfected with Myc-tagged KSR1 constructs (F, Δ328–392 or Δ319–433) or not transfected (−). Cells were lysed in buffer containing Ca^2+^ and complexes were immunoprecipitated (IP) with anti-Myc Affinity Gel. Samples were resolved by western blotting and probed with anti-Myc and anti-calmodulin antibodies. Lysates not subjected to immunoprecipitation were processed in parallel (Lysate). Data are representative of two independent experiments. The positions of migration of molecular mass markers are indicated on the *left* of the blots and gels.

The region on KSR1 to which calmodulin binds was investigated using selected fragments of KSR1. We generated GST fusion constructs of portions of KSR1, termed KSR1-N, KSR1-M, and KSR1-C (Fig. 3*A*). Each construct was incubated with pure calmodulin and the GST-tagged KSR1 fragments were isolated with glutathione-Sepharose. Samples were processed by SDS-PAGE and western blotting. The blots revealed that calmodulin bound to the M-fragment (amino acid residues 319–433) of KSR1 (Fig. 3*B*). By contrast, neither the N- (amino acid residues 2–318) nor the C-portion (amino acid residues 435–873) of KSR1 interacted with calmodulin.

In order to confirm the binding site, we generated deletion mutants of KSR1. Amino acids 319 to 433 and 328–392 were deleted from KSR1 (the constructs are termed KSR1Δ319–433 and KSR1Δ328–392, respectively). These plasmids, which are tagged with myc, were transiently transfected into HEK293 cells. Their ability to bind endogenous calmodulin in cells was evaluated by immunoprecipitating the KSR1 constructs with anti-Myc Affinity Gel. Myc-tagged full-length KSR1 was processed in parallel. Calmodulin co-immunoprecipitated with full-length KSR1 (Fig. 3*C*). By contrast, calmodulin failed to interact with either KSR1Δ319–433 or KSR1Δ328–392. These data show that amino acid residues 328–392 of KSR1 are necessary for binding cellular calmodulin and amino acids 319 to 433 are sufficient for *in vitro* calmodulin binding.

### Calmodulin influences EGF-induced activation of ERK

**Figure 4:**
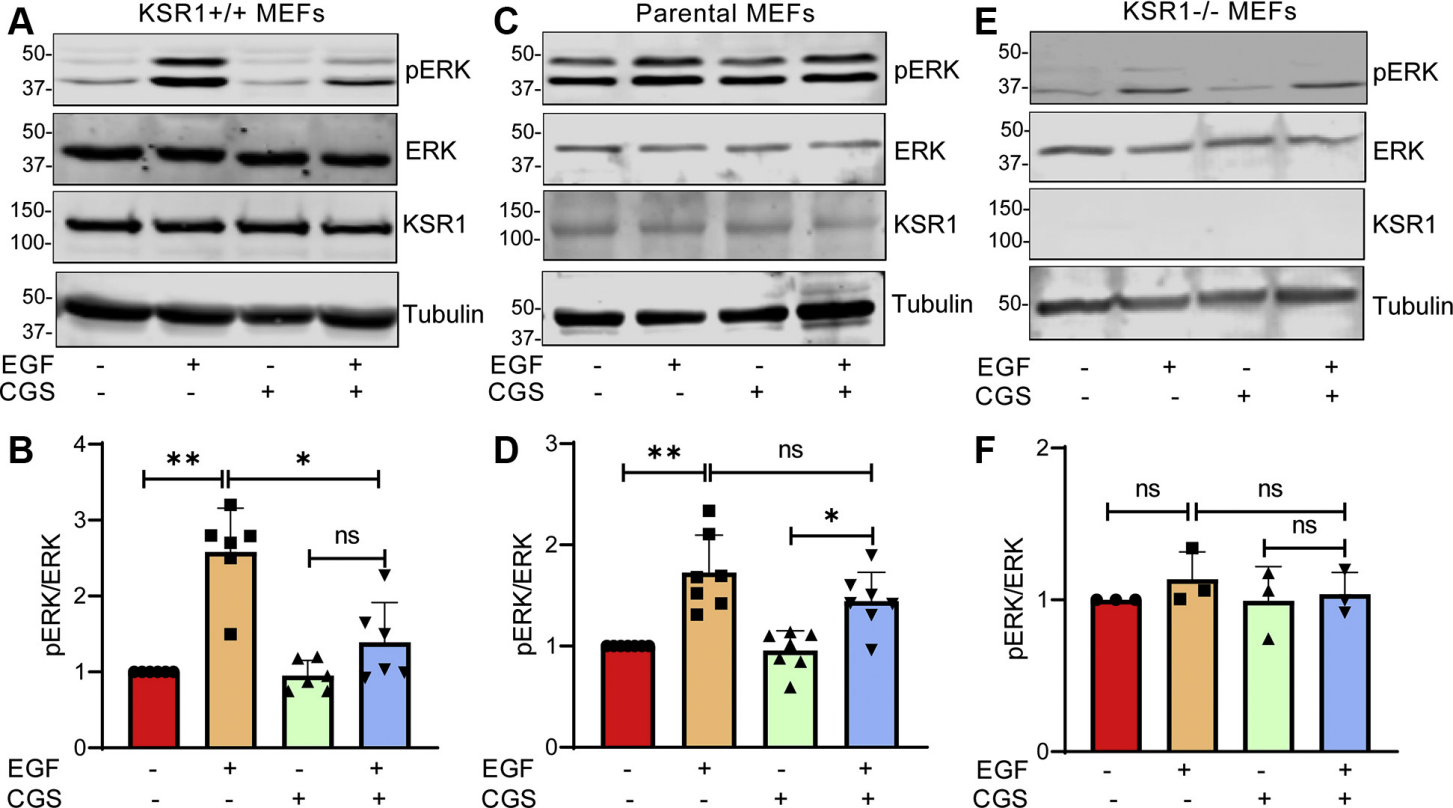
**Calmodulin influences EGF-induced activation of ERK.** KSR1+/+ MEFs (panels *A* and *B*), parental MEFs (panels *C* and *D*), and KSR1-null MEFs (panels *E* and *F*) were cultured in serum-free medium. Vehicle (−) or 40 μM CGS9343B (+) was added for 16 h, followed by the addition of vehicle (−) or 100 ng/ml EGF (+) for 10 min. Cells were lysed, samples were resolved by western blotting, and blots were probed with antibodies to phosphorylated ERK (pERK), total ERK, KSR1, and β-tubulin. The data are representative of at least three independent experiments. *B*, *D*, and *F*, pERK and total ERK bands were quantified with Image Studio 2.0 (LI-COR Biosciences). Graphs depict the ratio of pERK to total ERK in the same sample, with vehicle-treated samples (no CGS9343B or EGF) set as 1.0. Data represent means ± SD of 3 to 7 experiments. ∗*p* < 0.001; ∗∗*p* < 0.0001 using one-way ANOVA, with Tukey’s post-hoc test. ns, not significant.

To examine whether the interaction with calmodulin alters KSR1 function, we used the selective, cell-permeable calmodulin antagonist CGS9343B (29). We evaluated the effect of CGS9343B on activation of MAPK signaling induced by EGF in cells with different amounts of KSR1. Initially we preincubated serum-starved KSR1+/+ MEFs with CGS9343B or vehicle before adding EGF. Cell lysates were resolved by western blotting and probed with an antibody specific for the phosphorylated (activated) forms of ERK. In the absence of CGS9343B, EGF increased phosphorylated ERK (pERK) by 2.8- ± 0.29-fold (mean ± SD) in cells that overexpress KSR1 (Fig. 4, *A* and *B*). CGS9343B did not alter basal ERK phosphorylation. Notably, inhibiting calmodulin function with CGS9343B significantly reduced the ability of EGF to activate ERK; EGF enhanced phosphorylation of ERK by only 1.14- ± 0.33-fold (mean ± SD, *n* = 6), which was not significantly greater than that in unstimulated cells (Fig. 4, *A* and *B*). These results suggest that calmodulin function is required for maximal activation of the MAPK cascade by EGF in cells that overexpress KSR1. We established that the concentration of CGS9343B used in our assays was not toxic to the cells (data not shown).

To ascertain whether the inhibitory effect of CGS9343B on MAPK activation is mediated *via* KSR1, we investigated the effect of CGS9343B on EGF-induced ERK phosphorylation in cells that do not overexpress KSR1. In parental MEFs, EGF increased ERK phosphorylation by 1.7- ± 0.34-fold (mean ± SD) (Fig. 4, *C* and *D*). Importantly, the ability of EGF to promote ERK phosphorylation in control MEFs was not significantly impaired by CGS9343B; the increase was 1.4- ± 0.26-fold (mean ± SD, *n* = 7), which was significantly greater than that in unstimulated cells.

In order to unequivocally establish that the inhibitory effect of CGS9343B on ERK activation was mediated *via* KSR1, we used cells lacking KSR1. However, EGF failed to stimulate ERK phosphorylation in KSR1-null MEFs (Fig. 4, *E* and *F*), which is consistent with published data from others (13). Therefore, we are unable to use these cells to evaluate the potential effect of CGS9343B on MAPK activation. Nevertheless, the different effects we observed in KSR1+/+ and control MEFs strongly suggest that the inability of EGF to activate MAPK when calmodulin is inhibited is mediated *via* KSR1.

### CGS9343B prevents EGF from activating ERK at the plasma membrane

**Figure 5:**
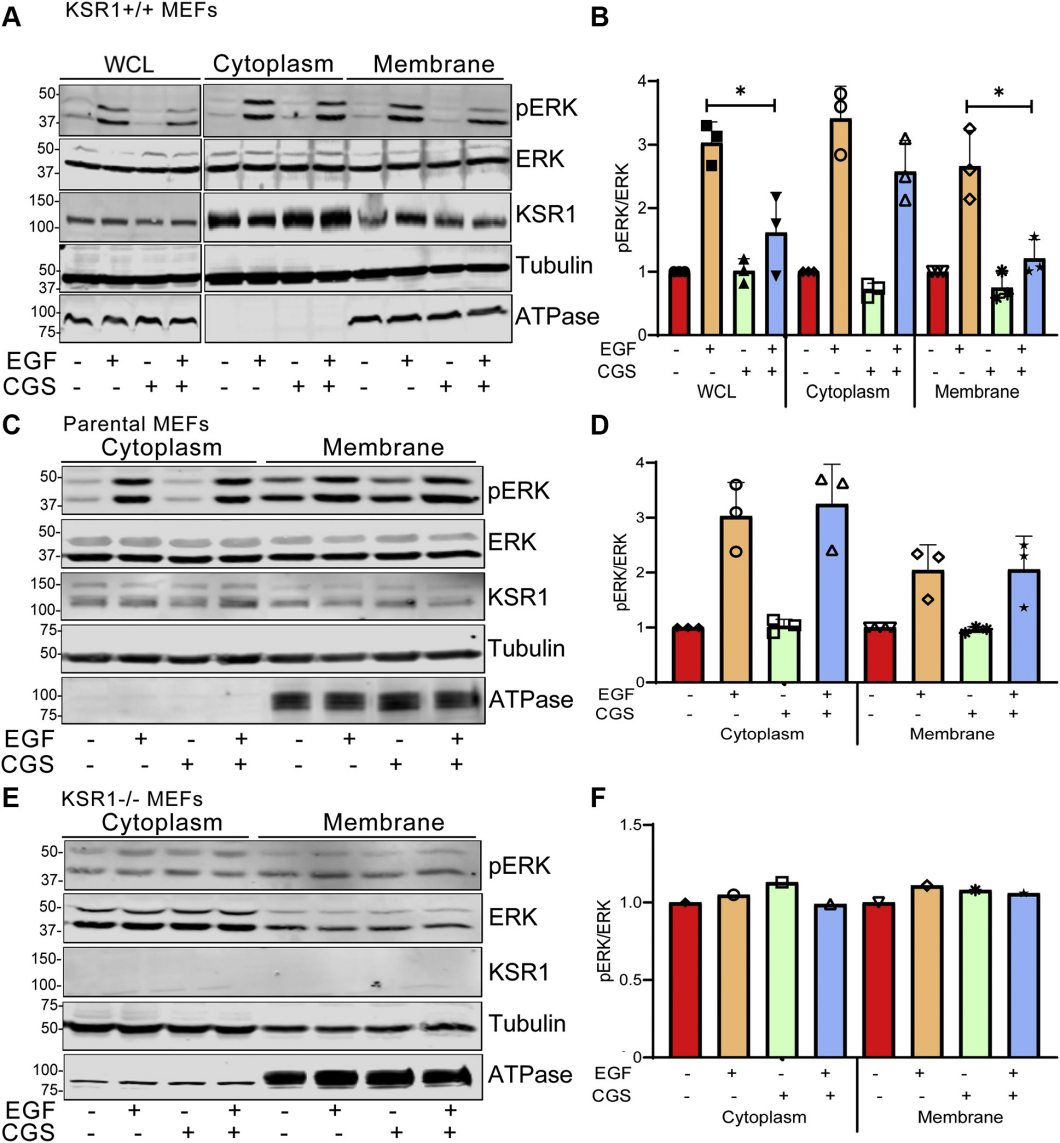
**CGS9343B prevents EGF from activating ERK at the plasma membrane.** Serum-starved KSR1+/+ MEFs (panels *A* and *B*), parental MEFs (*C* and *D*), and KSR1−/− MEFs (*E* and *F*) were pretreated with DMSO (−) or 40 μM CGS9343 (CGS, +) for 16 h, followed by incubation with vehicle (−) or 100 ng/ml EGF (+) for 5 min. Cell lysates were separated into cytoplasmic and membrane fractions as described under Experimental procedures. In total, 10 μl of whole-cell lysate (WCL; panel *A*, only), cytoplasmic and membrane fractions were resolved by SDS-PAGE and transferred to PVDF membranes. Blots were probed with anti-pERK, anti-ERK, anti-KSR1, anti-β-tubulin, and anti-Na^+^K^+^ ATPase antibodies. Na^+^K^+^ ATPase is the control for membrane fractions. Data in panels *A* and *C* are representative of three independent experiments and data in panel *E* are from a single experiment. *B*, *D*, and *F*, phosphorylation of ERK was quantified by densitometry using Image Studio 2.0 (LI-COR) and corrected for the amount of total ERK in the same sample. Data represent the means ± SD (panels *B* and *D*, *n* = 3 independent experiments) or a single value (panel *F*), with vehicle-treated cells set as 1.0. ∗*p* < 0.001 using one-way ANOVA, with Tukey’s post-hoc test.

Our next objective was to investigate the mechanism by which calmodulin modulates activation of MAPK by KSR1. EGF induces the translocation of KSR1 from the cytoplasm to the plasma membrane where it colocalizes with pERK (16). Two complementary approaches were used to assess the effect of calmodulin on the subcellular localization of KSR1 and pERK in response to EGF. In the first, we used subcellular fractionation to assess pERK localization at the plasma membrane upon EGF stimulation. After preincubation with vehicle or CGS9343B, cells were incubated with or without EGF, then separated into cytoplasmic and membrane fractions. Localization of pERK was examined by western blotting. In KSR1+/+ whole-cell lysates, EGF stimulated phosphorylation of ERK by 3.03- ± 0.32-fold (mean ± SD) (Fig. 5, *A* and *B*). Pretreatment with CGS9343B inhibited EGF-induced phosphorylation of ERK in whole-cell lysates by 46.8%. Analysis of fractions from vehicle-treated cells revealed that EGF increased pERK in the cytoplasmic and membrane fractions by 3.41- ± 0.5-fold (mean ± SD) and 2.66- ± 0.5-fold (mean ± SD), respectively (Fig. 5, *A* and *B*). Inhibition of calmodulin did not significantly reduce the ability of EGF to stimulate pERK in the cytoplasm. By contrast, CGS9343B completely prevented EGF from enhancing the membrane-bound pool of pERK (Fig. 5, *A* and *B*).

The effects of EGF and CGS9343B on MAPK activation were also investigated in cells that do not overexpress KSR1 (parental MEFs) and in cells lacking KSR1 (KSR1−/− MEFs). In parental MEFs, EGF slightly increased ERK phosphorylation in membrane fractions, but this was not statistically significant (Fig. 5, *C* and *D*). Consistent with the findings with total pERK in the cells in Figure 4, *C* and *D*, CGS9343B did not significantly impair the ability of EGF to promote ERK phosphorylation in membrane fractions of control MEFs (Fig. 5, *C* and *D*). The effect of EGF on pERK at the membrane could not be evaluated in KSR1−/− cells as EGF failed to stimulate ERK phosphorylation (Fig. 5, *E* and *F*). This observation is consistent with data from both this work (Fig. 4, *E* and *F*) and from others (13).

### Inhibiting calmodulin attenuates EGF-induced translocation of KSR1 to the plasma membrane

**Figure 6:**
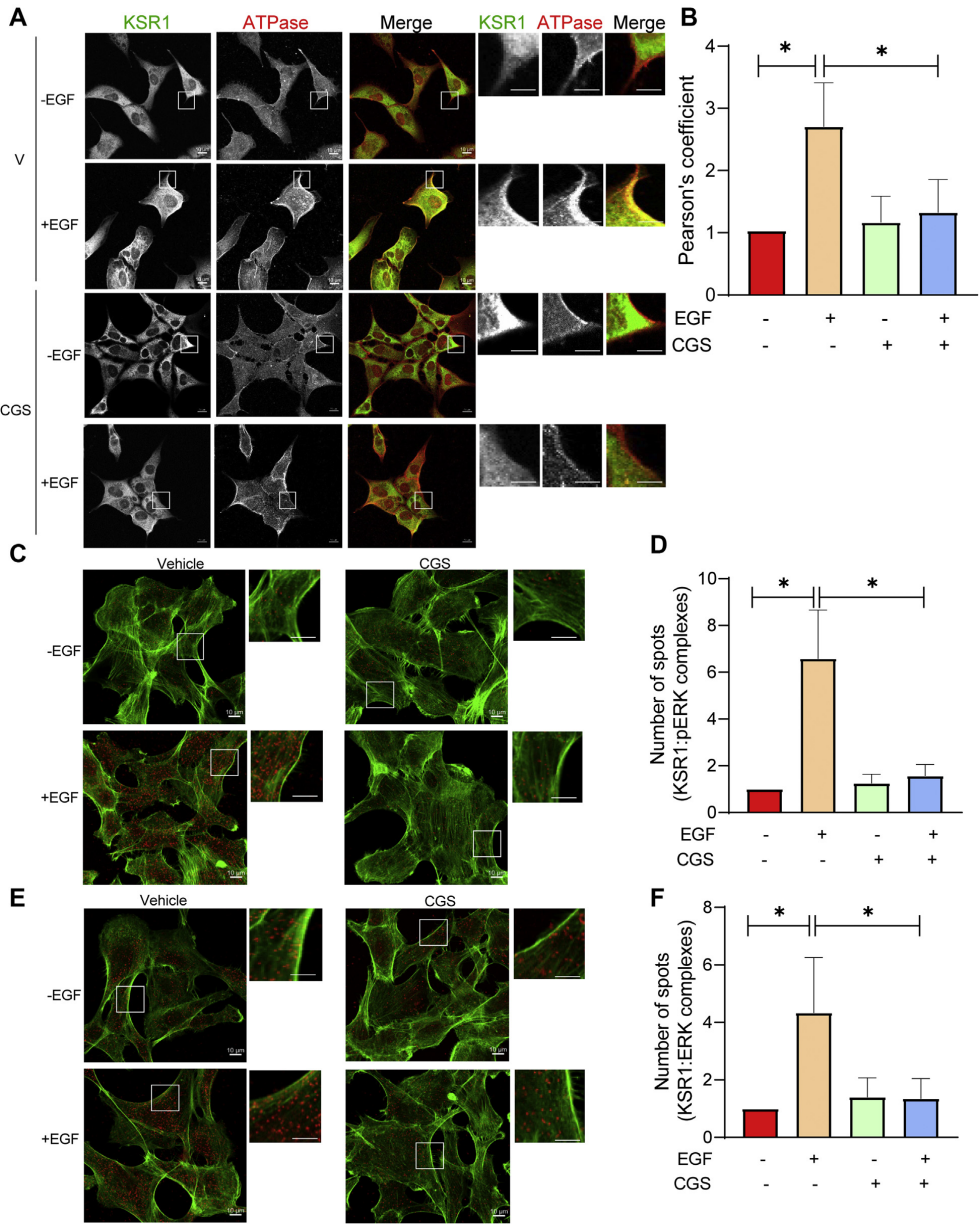
**Inhibiting calmodulin attenuates EGF-induced translocation of KSR1 to the plasma membrane and its association with ERK and pERK.***A*, serum-starved KSR1+/+ MEFs were pretreated with DMSO (V) or 40 μM CGS9343B (CGS). After 16 h, cells were incubated without (−) or with (+) 100 ng/ml EGF for 5 min. Cells were fixed, then probed with anti-KSR1 and anti-Na^+^K^+^ ATPase antibodies. Following incubation with the appropriate fluorescent-conjugated secondary antibodies, images were analyzed by Zeiss LSM780 confocal microscopy. The smaller panels on the right side are higher power magnifications of the areas encompassed by the *white boxes* on the images on the *left*. Merge is a composite of both channels; KSR1 is *green*, Na^+^K^+^ ATPase is *red*, and *yellow* indicates colocalization. Scale bars: *full panels*, 10 μm; *insets*, 3 μm. The data are representative of 50 cells for each condition. Negative controls showed no evidence of nonspecific staining or bleed-through. *B*, Pearson’s correlation coefficient was determined for colocalization of KSR1 and Na^+^K^+^ ATPase with Zen software. The data are expressed as means ± SD (n = 50 cells), with vehicle-treated cells set as 1. ∗*p* < 0.0001 using one-way ANOVA, with Tukey’s post-hoc test. *C* and *E*, KSR1+/+ MEFs were treated as described for panel *A*, then stained with both anti-KSR1 and anti-pERK (*C*) or anti-KSR1 and anti-ERK (*E*) antibodies. PLA was performed using Duolink detection reagents as described under Experimental procedures. *Red spots* indicate positive PLA. Actin was stained with Alexa Fluor 488 phalloidin (*green*). Representative images of 50 cells for each condition are shown. The *smaller panels* on the *right side* are higher power magnifications of the areas encompassed by the *white boxes* on the images on the *left*. Scale bar, *full panels*, 10 μm; *insets*, 3 μ. *D* and *F*, the number of PLA spots per cell was quantified with Image J software from confocal images of 50 cells for each condition. The data are expressed as means ± S.D. (error bars), with the number of spots in vehicle-treated cells set to 1. ∗*p* < 0.0001 by one-way ANOVA, with Tukey’s post-hoc test.

In the second approach, we used confocal microscopy. KSR1+/+ cells were preincubated with vehicle or CGS9343B, then stimulated with or without EGF. After 5 min, cells were fixed and stained with antibodies to KSR1 and to Na^+^K^+^ ATPase, a plasma membrane marker (30). Immunofluorescence confocal microscopy revealed that KSR1 was predominantly cytoplasmic in quiescent cells (Fig. 6*A*). Incubation with CGS9343B did not alter the cytoplasmic distribution of KSR1 in unstimulated cells. EGF induced translocation of KSR1 from the cytosol to the plasma membrane, where it colocalized with Na^+^K^+^ ATPase (Fig. 6*A*). Quantification of colocalization (Pearson’s coefficient) revealed that EGF significantly increased by 2.68- ± 0.19-fold (mean ± SD) the amount of KSR1 at the plasma membrane (Fig. 6*B*). Preincubating cells with the calmodulin antagonist CGS9343B completely prevented EGF from enhancing KSR1 translocation to the plasma membrane (Fig. 6, *A* and *B*).

Binding of KSR1 to ERK is essential for EGF-induced MAPK activation (11). The effect of calmodulin antagonism on the interaction between endogenous KSR1 and ERK was analyzed by the proximity ligation assay (PLA). In this technique, cells are incubated with specific antibodies and a signal is generated only when the antibodies are in close proximity (nm range). We preincubated serum-starved KSR1+/+ MEFs with CGS9343B or vehicle before adding EGF. PLA analysis with anti-KSR1 and anti-pERK antibodies demonstrated that EGF increased the interaction between KSR1 and pERK by 6.2- ± 0.37-fold (Fig. 6, *C* and *D*). CGS9343B did not alter KSR1-pERK in basal cells. Consistent with our observations that antagonizing calmodulin prevents EGF from stimulating translocation of KSR1 to the plasma membrane (Fig. 6, *A* and *B*), EGF was unable to enhance the formation of complexes between KSR1 and pERK in cells preincubated with CGS9343B (Fig. 6, *C* and *D*).

Analogous experiments were performed to examine KSR1 complex formation with total ERK. EGF increased the number of KSR1-ERK complexes (Fig. 6*E*), but the magnitude of increase (4.36- ± 0.41-fold) (Fig. 6*F*) was less than that between KSR1 and pERK. Inhibiting calmodulin with CGS9343B abrogated the ability of EGF to promote the formation of KSR1-ERK complexes (Fig. 6, *E* and *F*). Collectively, these data strongly support the hypothesis that calmodulin influences the ability of EGF to promote the scaffolding function of KSR1 in the MAPK cascade.

### EGF modulates the interaction between KSR1 and calmodulin

**Figure 7:**
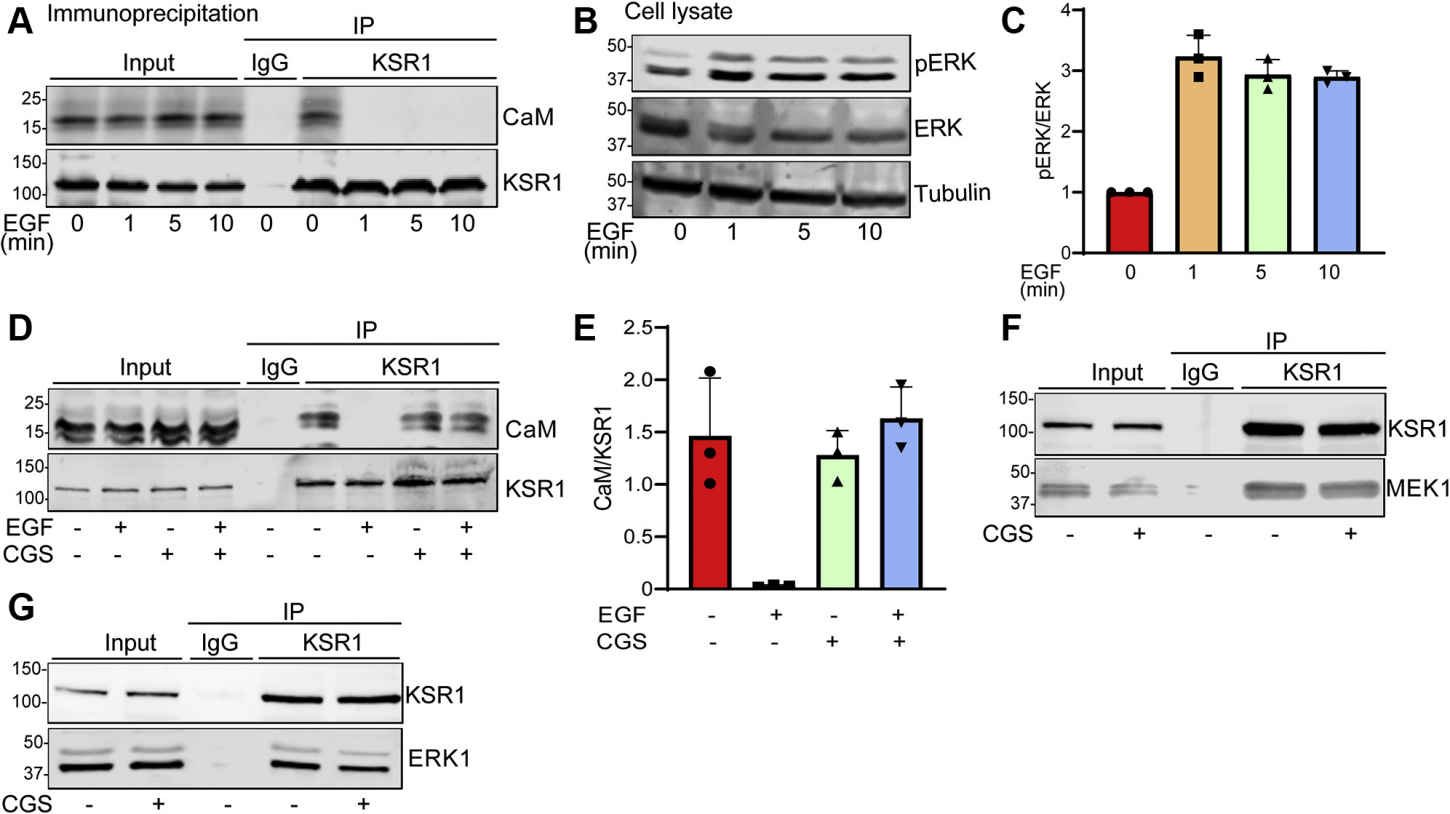
**EGF modulates the interaction between KSR1 and calmodulin.***A*, KSR1+/+ MEFs were serum-starved for 16 h, followed by incubation with 100 ng/ml EGF for 0, 1, 5, or 10 min. Cells were lysed in buffer containing 1 mM Ca^2+^ and 1 mg protein lysate was immunoprecipitated with anti-KSR1 antibody. A sample precipitated with mouse IgG was processed in parallel as negative control. Both unprocessed lysate (Input) and immunoprecipitates (IP) were resolved by western blotting and blots were probed with anti-calmodulin (CaM) and anti-KSR1 antibodies. *B*, equal aliquots of protein from the cell lysates used for immunoprecipitation were resolved by western blotting and probed with antibodies to phosphorylated ERK (pERK), total ERK, and β-tubulin (loading control). *C*, pERK and total ERK bands were quantified with Image Studio 2.0 (LI-COR Biosciences). Graphs depict the ratio of pERK to total ERK in the same sample, with vehicle-treated samples set as 1.0. Data represent means ± SD of three independent experiments. *D*, serum-starved cells were incubated with vehicle (−) or 40 μM CGS9343B (CGS, +). After 16 h, DMSO (−) or 100 ng/ml EGF (+) was added for 10 min. Samples were processed as described for panel *A*. All images are representative of three independent experiments. *E*, the calmodulin and KSR1 bands were quantified with Image Studio 2.0 (LI-COR Biosciences). Graphs depict the ratio of calmodulin (CaM) to KSR1 in the same sample. Data represent means ± SD of three independent experiments. *F*, serum-starved KSR1+/+ MEFs were incubated with vehicle (−) or 40 μM CGS9343B (CGS, +) for 16 h. Cells were lysed and 1 mg protein lysate was immunoprecipitated with anti-KSR1 antibody. A sample precipitated with mouse IgG was processed in parallel as negative control. Both unprocessed lysate (Input) and immunoprecipitates (IP) were resolved by western blotting and blots were probed with anti-KSR1 and anti-MEK1 antibodies. *G*, cells were treated as described for panel *F*, except the blots were probed with anti-KSR1 and anti-ERK antibodies. All data are representative of three independent experiments.

As EGF activates the MAPK cascade (2), we examined whether EGF modulates the interaction between KSR1 and calmodulin. Cells were serum-starved for 16 h, then incubated with EGF for 0, 1, 5, or 10 min. After lysis in buffer containing Ca^2+^, samples were immunoprecipitated with anti-KSR1 antibody. As shown above in Figure 2, calmodulin co-immunoprecipitated with KSR1 from serum-starved cells (Fig. 7*A*). Surprisingly, EGF completely abrogated the interaction between KSR1 and calmodulin at all the time points evaluated (Fig. 7*A*). In order to confirm that EGF activated MAPK signaling under our experimental conditions, we assessed the amount of pERK in the cells. EGF induced a threefold increase in pERK at 1 min, and this effect persisted for 10 min, validating activation of the MAPK cascade (Fig. 7, *B* and *C*). Our results demonstrate that EGF induces the dissociation of KSR1 and Ca^2+^/calmodulin.

### CGS9343B prevents EGF from disrupting the interaction between KSR1 and calmodulin

In order to gain further insight into the molecular mechanism by which inhibition of calmodulin impairs the ability of KSR1 to mediate MAPK activation by EGF, we evaluated the effect of CGS9343B on the binding of calmodulin to KSR1 in cells. We pretreated cells with CGS9343B for 16 h prior to analysis of the interaction between the proteins. Inhibition of calmodulin had no effect on the amount of calmodulin that co-immunoprecipitated with KSR1 from serum-starved cells (Fig. 7, *D* and *E*). Importantly, when cells were preincubated with CGS9343B, EGF failed to disrupt the binding between calmodulin and KSR1. The amount of calmodulin that co-immunoprecipitated with KSR1 from cells treated with both CGS9343B and EGF was the same as that from serum-starved cells (Fig. 7, *D* and *E*). Note that CGS9343B did not significantly change the amounts of calmodulin or KSR1 in the cells (Fig. 7*D*, input). Taken together, these data reveal that Ca^2+^/calmodulin binds constitutively to KSR1 in cells. EGF rapidly induces dissociation, but antagonism of calmodulin completely prevents EGF from uncoupling the interaction between KSR1 and calmodulin.

We next evaluated whether CGS9343B had any effect on the interaction of KSR1 with its well-characterized binding partners MEK1 and ERK. KSR1+/+ MEFs were incubated with CGS9343B or vehicle. The binding between KSR1 and MEK1 as well as between KSR1 and ERK was evaluated by immunoprecipitating KSR1 from cell lysates and probing western blots for MEK1 or ERK. The amount of endogenous MEK1 that co-immunoprecipitated with KSR1 was essentially the same in the presence as in the absence of CGS9343B (Fig. 7*F*). Similarly, CGS9343B did not significantly alter the amount of ERK that co-immunoprecipitated with KSR1 from serum-starved cells (Fig. 7*G*). The absence of MEK1 and ERK from the samples precipitated with IgG validates the specificity of the interactions. Collectively, these data show that the effect of CGS9343B on KSR1 function is mediated *via* its antagonism of calmodulin.

## Discussion

KSR1 was identified 25 years ago as a modulator of Ras signaling and later was shown to be a scaffold for the Raf/MEK/ERK cascade (31). The cellular activities in which KSR1 participates range from cell proliferation and survival to glucose metabolism and adipogenesis (32). Notwithstanding multiple publications from numerous investigators, the mechanisms by which KSR1 modulates MAPK signaling to elicit these effects remain incompletely understood.

Ca^2+^ and calmodulin are known to influence MAPK signaling [for reviews, see Refs. (20, 21)], but the effects are complex and highly variable. Most studies have shown that increased intracellular free Ca^2+^ concentration ([Ca^2+^]_i_) activates the ERK cascade in neuronal cells (33). An increase in [Ca^2+^]_i_ in neurons induces ERK phosphorylation independently of NGF (34). Moreover, intracellular Ca^2+^ and calmodulin are both required for NGF to stimulate ERK activity (21). By contrast, Ca^2+^-inhibited ERK activation by EGF in keratinocytes (35) and chelation of intracellular Ca^2+^ in fibroblasts led to an increase in ERK activation (36, 37). These effects are mediated *via* several Ca^2+^-modulated enzymes, including PYK2 (38), protein kinase C (39), or RasGRF (22). There are substantial discrepancies in the published literature regarding the role of calmodulin in MAPK signaling. The calmodulin antagonist W13 blocked phosphorylation of ERK induced by NGF in PC12 cells (40), but augmented activation of ERK by EGF in Swiss 3T3 cells (23). In COS-1 cells, inhibition of calmodulin reduced the ability of EGF to activate MAPK signaling (41). W13 was reported to increase ERK phosphorylation in serum-starved NIH 3T3 fibroblasts, but had no effect on activation of ERK at 10 or 30 min after serum stimulation (42). By contrast, inhibition of calmodulin blocks induction of ERK by gonadotropin-releasing hormone (43). The reasons for the variation among the studies are not known, but differences in experimental conditions and cell types presumably contribute. An important limitation of the literature is that very few studies provide insight into the mechanism by which calmodulin influences MAPK signaling.

In this study, we describe a previously unidentified association of calmodulin with the scaffold protein KSR1 and explore the effects on MAPK signaling. We demonstrate direct binding of KSR1 to calmodulin by *in vitro* analysis with pure proteins. The binding is regulated by - and dependent on - Ca^2+^ as chelation of Ca^2+^ with EGTA completely abrogated the interaction. We observed similar regulation by Ca^2+^ in cell lysates; KSR1 and endogenous calmodulin co-immunoprecipitated only when Ca^2+^ was present. Ca^2+^ regulates the interaction of calmodulin with numerous target proteins. While apocalmodulin (Ca^2+^-free calmodulin) can bind to some proteins (44), particularly those with IQ motifs (45), Ca^2+^/calmodulin binds to a far greater number of targets (19). On binding Ca^2+^, calmodulin undergoes considerable conformational change, allowing it to associate with and regulate myriad target proteins, ranging from kinases and phosphatases to channels and nuclear receptors (46). In addition, more recent evidence reveals that calmodulin can also bind directly to scaffold proteins, such as IQGAP1, AKAP, Grb7, insulin receptor substrate, and striatin (47). Determining the functional role of this interaction is difficult (47), but has been achieved for a few scaffolds (48, 49, 50).

To ascertain whether calmodulin influences KSR1 function in cells, we adopted the very widely used strategy of inhibiting calmodulin function with a cell-permeable chemical antagonist. We selected CGS9343B since it is specific for calmodulin at concentrations of up to 1000 μM (29) and has been used by both our group (51, 52) and others (53) to block intracellular calmodulin function. In order to minimize the possibility of a nonspecific effect of the inhibitor, we used a low concentration of CGS9343B (40 μM), which is only 4% of the concentration up to which it has been demonstrated to be specific (29). We evaluated the effect of CGS9343B on KSR1 scaffolding in MAPK signaling. When the MAPK cascade was activated by EGF in cells that overexpress KSR1, CGS9343B abrogated the normal increase in ERK activity. By contrast, inhibition of calmodulin did not significantly reduce EGF-stimulated ERK activity in MEFs with less KSR1. The reason for the latter observation is not known. It is important to note that the KSR1+/+ cells that we used express KSR1 at a level that is optimal for signaling, having a markedly greater increase in EGF-stimulated activation of MAPK than that seen in cells with wild-type KSR1 expression levels (13). This is attributable to the finding that the KSR1 level for maximal signaling is coincident with the level of KSR1 expression that maximally associates with all the members of the RAF/MEK/ERK cascade (13). Increasing or reducing KSR1 beyond this level markedly reduces the formation of the complex of KSR1 with the MAPK components. For this reason, the overwhelming majority of the published studies on KSR1 use cells that are transfected with KSR1 (*e.g.*, Refs. (12, 15, 16, 54)). There are analogous observations of an optimal concentration of other MAPK scaffolds to facilitate activation of MEK and ERK by EGF (3, 8).

We gained further mechanistic insight into the role of calmodulin in KSR1 function by assessing the subcellular localization of KSR1. EGF stimulates translocation of KSR1 in a complex with MEK to the plasma membrane where it assembles a multiprotein complex in which Raf activates MEK, leading to ERK activation (2). When we interrogated this process, we observed by both confocal microscopy and subcellular fractionation that CGS9343B reduced EGF-stimulated translocation of KSR1 to the plasma membrane. In addition, we showed by PLA that the calmodulin antagonist abrogated the ability of EGF to promote the interaction of KSR1 with total ERK and with pERK in cells. Consistent with these observations, inhibition of calmodulin prevented EGF from activating ERK at the plasma membrane.

**Figure 8:**
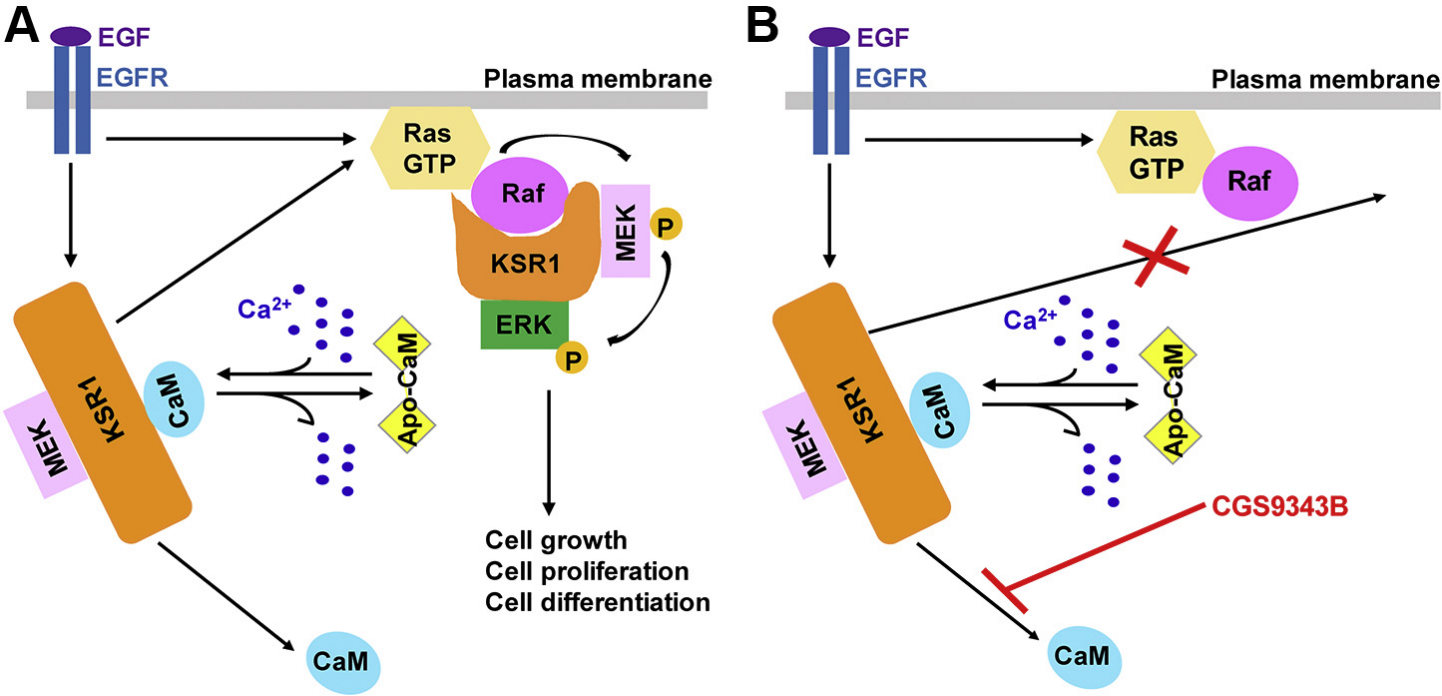
**Model depicting the interaction between Ca**^**2+**^**/calmodulin and KSR1 in MAPK signaling**. *A*, Ca^2+^/calmodulin binds constitutively to KSR1. Stimulation of EGFR induces both the activation of Ras at the plasma membrane and dissociation of Ca^2+^/calmodulin from KSR1. The latter enables KSR1 to translocate to the plasma membrane where it scaffolds Raf/MEK/ERK, enabling ERK activation. *B*, the calmodulin antagonist CGS9343B prevents the dissociation of Ca^2+^/calmodulin from KSR1. The Ca^2+^/calmodulin-KSR1 complex is unable to move to the plasma membrane, attenuating MAPK activation. Other proteins that bind KSR1 are not included for clarity. *CaM*, calmodulin; *EGFR*, EGF receptor; *P*, phosphate.

Another important observation we made is that EGF induces the dissociation of calmodulin from KSR1 and this effect is prevented by CGS9343B. The latter finding suggests that KSR1 cannot move to the membrane when bound to calmodulin. Based on our both data and observations by others, we propose a model for the cross talk between Ca^2+^/calmodulin and KSR1 in MAPK signaling (Fig. 8). In quiescent cells, Ca^2+^/calmodulin is constitutively bound to KSR1. When EGF binds to and activates the EGF receptor, Ca^2+^/calmodulin dissociates from KSR1, which enables KSR1 to move to the plasma membrane. EGF also activates Ras at the membrane, enabling KSR1 to assemble a complex of Raf/MEK/ERK and facilitate sequential phosphorylation for ERK activation. The active ERK then promotes selected biological functions (Fig. 8*A*). Antagonism of calmodulin prevents EGF from inducing the dissociation of Ca^2+^/calmodulin from KSR1, which prevents KSR1 from translocating from the cytoplasm to the plasma membrane (Fig. 8*B*). Thus, KSR1 is unable to facilitate activation of the MAPK cascade by EGF when cells are incubated with CGS9343B. In this model, calmodulin acts as a negative regulator that keeps KSR1 in the “off” state by preventing KSR1 from activating MAPK signaling in cells under basal, unstimulated conditions. It is tempting to speculate that certain pathophysiological conditions (*e.g.*, carcinoma) may dissociate calmodulin from KSR1, enabling KSR1 to scaffold and activate MAPK in the absence of EGF or possibly other activators.

The detailed molecular mechanism by which calmodulin influences KSR1 translocation to the plasma membrane is unknown. This is due, at least in part, to a lack of comprehension of the mechanism by which KSR1 trafficks from the cytoplasm to the membrane. Nevertheless, there are several possibilities. When calmodulin binds to a target protein, it frequently alters the tertiary conformation of the target protein (55). It is feasible that calmodulin binding modifies the conformation of KSR1, which alters the interaction of KSR1 with (an)other protein(s). For example, Ca^2+^/calmodulin could increase the association of protein 14-3-3 with KSR1, which sequesters it in the cytosol. A second possibility is that the conformation adopted by KSR1 in a complex with calmodulin obscures its membrane-binding domain. KSR1 is targeted to the plasma membrane by its CC-SAM domain in growth factor-treated cells (54). Thirdly, calmodulin could directly modulate the subcellular localization of KSR1. Numerous publications have documented that calmodulin regulates movement of proteins within the cell. For example, analogous to our finding with KSR1 here, chemical antagonism of calmodulin reduced the ability of insulin to induce translocation of GLUT4 from the cytosol to the plasma membrane (56). Calmodulin sequesters c-rel in the cytosol and a calmodulin inhibitor induces translocation of c-rel into the nucleus (57). Importantly, calmodulin also influences the subcellular localization of another MAPK scaffold. CGS9343B significantly increased the amount of IQGAP1 at areas of MCF-7 epithelial cell–cell contact (24). Ca^2+^/calmodulin triggers the release of IQGAP1 from actin filaments, which causes IQGAP1 to dissociate from the cell cortex (26). Moreover, localization to the leading edge of migrating cells of a point mutant IQGAP1 construct that specifically lacks binding to Ca^2+^/calmodulin is 50% of that of wild-type IQGAP1 (50). The possibilities mentioned here are not mutually exclusive and more than one may contribute. Regardless of the mechanism, our data suggest that calmodulin influences the ability of KSR1 to maximally activate ERK at the plasma membrane.

While intracellular signaling pathways were initially thought to be simple linear processes, it subsequently became clear that there is cross talk between different signaling cascades and interconnections can occur at many different points (58). Cross talk from one signaling cascade results in alterations in the activity of another. In this study we describe for the first time an intersection between the Ca^2+^ signaling and MAPK pathways *via* a direct interaction between Ca^2+^/calmodulin and KSR1. These observations expand our comprehension of intracellular signaling networks. Moreover, since normal MAPK homeostasis is disrupted in several human diseases, ranging from cancer (32) and developmental disorders (59) to Alzheimer’s disease (60) and age-related macular degeneration (61), our findings provide an additional avenue of exploration to develop new therapeutic approaches to ameliorate these conditions.

## Experimental procedures

### Materials

Dulbecco’s modified Eagle’s medium (DMEM), fetal bovine serum (FBS), Dynal-beads protein A/G, Coomassie brilliant blue R-250 stain, and paraformaldehyde were purchased from Thermo Scientific. Glutathione-Sepharose (17-5132-01), calmodulin-Sepharose (17-0529-01), and protein A-Sepharose (17-0780-01) beads were from GE Healthcare. Pure calmodulin was obtained from Ocean Biologics. Anti-β-tubulin antibody (T8328), EZview Red Anti-c-Myc Affinity Gel (E6654), EGF (SRP3027), CGS9343B (C1619), and Immobilon-FL polyvinylidene difluoride (PVDF) membrane (IPFL00010) were purchased from Sigma-Aldrich. The anti-calmodulin monoclonal antibody has been characterized (28). InstantBlue Protein stain (ISB1L) was purchased from Expedeon. Blocking buffer (927-80001) and IR dye-conjugated (IRDye) secondary antibodies (anti-rabbit 926-32213, anti-mouse 926-68022) were purchased from LI-COR Biosciences. Antibodies and dilutions are listed in Table 1.

**Table 1:** Antibodies used in this study

Protein detected	Reference	Dilution for immunoblots
Calmodulin	Monoclonal antibody (28)	1:1000
ERK1/2	Cell Signaling, 9107S	1:1000 (immunoblots) 1:100 (PLA)
GST	Santa Cruz, sc-138	1:1000
KSR1	Abcam, ab68483	1:1000 (immunoblots)
KSR1	Santa Cruz, sc-515924	3 μg (immunoprecipitation) 1:100 (PLA) 1:100 (immunostaining)
MEK1	Cell Signaling, 9122S	1:1000
Myc	Sigma, 06-549	1:1000
Na^+^K^+^ ATPase	Cell Signaling, 3010S Abcam, ab76020	1:1000 (immunoblots) 1:100 (immunostaining)
pERK1/2	Cell Signaling, 4377S	1:1000 (immunoblots) 1:100 (PLA)
Tubulin	Sigma, T5201	1:1000

### Plasmid construction

To generate the pGEX2T-TEV-KSR1-N and -M plasmids, PCR was performed using pTrace-His-KSR1 as a template with 5’-CGAGATCTGATAGAGCGGCGTTGCGCGCGGCA-3’ (forward) and 5’- CGGAATTCTCTAGACTACGTGGTGGAGGATGGGTTGCTGC-3’ (reverse) primers. The PCR product of base pairs 2 to 433 of KSR1 was digested with BglII, BamHI, and EcoRI and the fragments were purified. To obtain pGEX2T-TEV-KSR1-N, the larger fragment (base pairs 2–318) was inserted into pGEX2T-TEV at BamHI site. pGEX2T-TEV-KSR1-M was obtained by inserting the smaller fragment (base pairs 319–433) into pGEX2T-TEV at BamHI and EcoRI sites. To generate the pGEX2T-TEV-KSR1-C plasmid, PCR was performed using pTrace-His-KSR1 as a template with 5’-CGAGATCTTCCTCCACACCCTCATCGCCGGCA-3’ (forward) and 5-CGGAATTCTCTAGACTACATCTTTGGATTACCGGACTCCA-3’ (reverse) primers. The PCR product of KSR1-C was cut with BglII and EcoRI and inserted into pGEX2T-TEV at BamHI and EcoRI sites.

To generate pcDNA3-myc-KSR1 (full length), PCR was performed using pTrace-His-KSR1 as a template with 5’-CGAGATCTGATAGAGCGGCGTTGCGCGCGGCA-3’ (forward) and 5’-CGGAATTCTCTAGACTACATCTTTGGATTACCGGACTCCA-3’ (reverse) primers. The PCR product of the full-length KSR1 was cut with BglII and XbaI, and the fragment was inserted into pcDNA3-myc at BamH1 and XbaI sites. pcDNA3-myc-KSR1Δ328–392 was generated by making a partial cut with EcoRV in the pcDNA3-myc-KSR1 construct. The 7900 bp fragment was purified and subjected to self-ligation. pcDNA3-myc-KSR1Δ319–433 was made in three sequential steps. (i) pBlueScriptII-KSR1 was made by digesting the PCR product of full-length KSR1 with ECoRI and inserting it into the pBlueScriptII vector at EcoR1 and EcoRV sites. (ii) pBlueScriptII-KSR1Δ319–433 was made using pBlueScriptII-KSR1 as template with 5’-TCCTCCACCACGTCCTCCACACCCTCATCG-3’ (forward) and 5’-ATGAGGGAGTTCAAACTTCATCGGGGTGAC-3’ (reverse). The PCR product was subjected to self-ligation. (iii) pcDNA3-myc-KSR1Δ319–433 was generated by digesting pBluescriptII-KSR1Δ319–433 with BglII and XbaI, and the purified fragments were inserted into pcDNA3-myc at BamHI and XbaI sites. The sequences of all plasmids were confirmed by DNA sequencing. Plasmids were purified with QIAprep Spin Mini Prep Kit (QIAGEN). All constructs migrated to the expected position on SDS-PAGE.

### Cell lines and culture conditions

KSR1+/+ and KSR1−/− mouse embryonic fibroblast (MEF) cells were kindly provided by Robert Lewis, University of Nebraska Medical Center (13). The level of KSR1 in KSR1+/+ MEFs is ninefold higher than that in control MEFs (13). MEFs and HEK-293 cells (purchased from ATCC) were grown at 37 °C and 5% CO_2_ in DMEM, supplemented with 10% FBS. Cells were plated in 100-mm (for immunoprecipitation) or 60-mm (for fractionation) dishes. After the cells attached, the medium was replaced with serum-free medium. Vehicle (dimethyl sulfoxide, DMSO) or 40 μM CGS9343B (calmodulin antagonist) was added, followed 16 h later by the addition of 100 ng/ml EGF or an equal volume of vehicle (DMSO) for the times indicated in the figure legends.

### Purification of His-KSR1 and GST-KSR1

pTrace-His-KSR1, kindly provided by Piero Crespo (Instituto de Biomedicina y Biotecnologia de Cantabria, Spain), was expressed in *E. coli*, which were grown in 4 l of Luria Bertani broth overnight at 25 °C. Iso-propyl-β-D-thiogalactoside (American Bio, catalog no. AB00841) was added at a final concentration of 20 μM and the cells were cultured for an additional 8 h at 25 °C, then harvested by centrifugation. The cell pellet was suspended in lysis buffer A (50 mM NaH_2_PO_4_, 300 mM NaCl, 10 mM imidazole, pH 8.0), followed by sonication and centrifugation. The supernatant was passed through TALON metal affinity resin column (635506, BD Biosciences) and washed with wash buffer (50 mM NaH_2_PO_4_, 300 mM NaCl, 20 mM imidazole, pH 8.0). Proteins bound to the column were eluted with elution buffer (50 mM NaH_2_PO_4_, 300 mM NaCl, 250 mM imidazole, pH 8.0) and dialyzed against PBS (phosphate buffered saline) containing 6 mM β-mercaptoethanol. His-KSR1 protein was 90% pure by Coomassie blue staining. GST-KSR1 constructs were expressed in *E. coli* and induced with 1.5 μM iso-propyl-β-D-thiogalactoside at 25 °C. Proteins were isolated with glutathione-Sepharose beads essentially as previously described (62).

### Binding assays

KSR1+/+ MEFs were lysed in 1 ml of lysis buffer B (50 mM Tris-HCl, pH 7.4, 150 mM NaCl, 1% Triton-X 100) containing Halt protease inhibitor cocktail (Thermo Fisher Scientific, catalog no. 78429) with 1 mM CaCl_2_ or 1 mM EGTA. One milligram of protein lysate was precleared with glutathione-Sepharose beads for 1 h at 4 °C, then incubated with 30 μl calmodulin-Sepharose or GST bound to glutathione-Sepharose beads (negative control) on a rotator at 4 °C for 3 h. Beads were washed five times in lysis buffer B containing 1 mM CaCl_2_ or 1 mM EGTA and resuspended in SDS-PAGE sample buffer (0.5 M Tris-HCl, pH 6.8, 3.8 g glycerol, 1 g SDS, 0.93 g dithiothreitol and 1.2 mg bromophenol blue). The samples were heated at 100 °C for 5 min, resolved by SDS-PAGE and transferred to PVDF membrane. The membranes were probed with anti-calmodulin and anti-KSR1 primary antibodies, followed by the appropriate IRDye-conjugated secondary antibodies. Blots were scanned using the Odyssey imaging system (LI-COR Biosciences).

Binding analyses of pure proteins were performed by incubating 500 ng purified His-KSR1 (precleared with glutathione-Sepharose beads for 1 h at 4 °C) with 30 μl calmodulin-Sepharose in 1 ml lysis buffer B containing 1 mM CaCl_2_ or 1 mM EGTA on a rotator for 3 h at 4 °C. GST-Sepharose was used as the negative control. Beads were washed five times as described above and subjected to western blotting with anti-KSR1 and anti-GST antibodies.

### Binding site analysis

Two micrograms of pure calmodulin in 1 ml of lysis buffer B containing 1 mM Ca^2+^ was precleared with glutathione-Sepharose beads for 1 h at 4 °C, followed by incubation with the GST-KSR1 constructs (KSR1-N, KSR1-M and KSR1-C) on glutathione-Sepharose beads for 3 h at 4 °C. After sedimentation by centrifugation, the beads were washed, and western blotting was performed as described above. The gel was cut at ∼25 kDa. The lower portion of the gel was subjected to western blotting and probed with anti-calmodulin antibody. The upper portion of the gel was stained with Coomassie blue.

The pcDNA3-myc-KSR1 plasmids, namely full-length KSR1, KSR1Δ328–392, and KSR1Δ319–433, were transfected into HEK-293 cells using Lipofectamine 2000 (Invitrogen) transfection reagent according to the manufacturer's instructions. Forty-eight hours after transfection, the cells were lysed in 1 ml lysis buffer B containing 1 mM CaCl_2_ and subjected to centrifugation at 15,000*g* for 5 min at 4 °C. The lysates were precleared with glutathione-Sepharose beads for 1 h at 4 °C, then incubated with anti-Myc Affinity Gel on a rotator for 3 h at 4 °C. After sedimentation by centrifugation, the beads were washed, and western blotting was performed as described above.

### Immunoprecipitation

KSR1+/+ MEFs were lysed in 1 ml lysis buffer B containing 1 mM CaCl_2_ or 1 mM EGTA and subjected to centrifugation at 15,000*g* for 5 min at 4 °C. The lysates were precleared with protein A-Sepharose beads for 1 h at 4 °C, then incubated with anti-mouse IgG (control) or 3 μg anti-KSR1 monoclonal antibody on a rotator for 3 h at 4 °C. Immune complexes were precipitated with 30 μl Dynal-beads protein A/G for 90 min. The beads were washed five times with lysis buffer B containing 1 mM Ca^2+^ or 1 mM EGTA, resuspended in SDS-PAGE sample buffer, heated at 95 °C for 5 min, and then analyzed by SDS-PAGE and western blotting. For calmodulin immunoprecipitation, anti-mouse IgG (control) or anti-calmodulin monoclonal antibody (28) was incubated with 30 μl protein A-Sepharose beads in 500 μl PBS for 2 h at 4 °C on a rotator. One milligram of precleared cell lysate prepared from KSR1+/+ MEFs in the presence of 1 mM Ca^2+^ or 1 mM EGTA was incubated with antibody-coated beads for 3 h at 4 °C on a rotator. After sedimentation by centrifugation, the beads were washed, and western blotting was performed as described above.

### Cell viability assay

KSR1+/+, KSR1−/−, and parental MEFs were added to a 96-well plate. After the cells attached, the medium was replaced with serum-free medium. Vehicle (DMSO) or 40 μM CGS9343B was added for 16 h and cell viability was measured using CellTiter 96 aqueous one solution cell proliferation assay (G3580, Promega), as recommended by the manufacturer. Briefly 20 μl reagent was added to 100 μl medium in each well and cells were incubated at 37 °C for 1 h. The absorbance was measured at 490 nM using a BioTek Synergy 4 microplate reader.

### Immunostaining

KSR1+/+ MEFs were plated on coverslips (Electron Microscopy Sciences, Cat # 72230-01) and incubated at 37 °C and 5% CO_2_ in DMEM, supplemented with 10% FBS. After the cells attached, the medium was replaced with serum-free medium. Vehicle (DMSO) or 40 μM CGS9343B (calmodulin antagonist) was added, followed 16 h later by the addition of 100 ng/ml EGF or vehicle for 5 min. The coverslips were washed with ice-cold PBS, then fixed in 3.75% (v/v) paraformaldehyde for 15 min at 22 °C. Cells were permeabilized in 0.25% (v/v) Triton-X 100 for 15 min and blocked for 1 h in 1% (w/v) bovine serum albumin at 22 °C. Cells were incubated with anti-KSR1 mouse monoclonal antibody and with anti-Na^+^K^+^ ATPase rabbit antibody for 16 h at 4 °C. (Cells incubated without the primary antibody served as the negative control.) Unbound antibody was removed by washing five times with PBS, followed by incubation for 2 h with Alexa Fluor 568-labeled anti-rabbit antibody (red; Invitrogen, A11036) and Alexa Fluor 488-labeled anti-mouse antibody (green; Invitrogen, A11029). After five washes with PBS, the cells were mounted with ProLong Glass Antifade mounting medium (Invitrogen, P36980). Cells were examined with a Zeiss LSM 780 confocal microscope using 63× objective lens. Fifty cells were examined for each condition. For quantification of the immunostained proteins, Pearson’s correlation coefficient for colocalization was determined for image pairs of KSR1 and Na^+^K^+^ ATPase using Zen software.

### Proximity ligation assay

PLA was performed essentially as previously described (63). Briefly, KSR1+/+ MEFs, plated on coverslips in 24-well plates, were fixed in 4% paraformaldehyde and then permeabilized with 0.25% Triton X-100. After blocking in 10% FBS for 2 h, slides were incubated overnight at 4 °C with anti-KSR1 antibodies and either anti-pERK or anti-ERK antibodies. Donkey anti-mouse PLUS (catalog no. DUO92001-30RXN Sigma) and donkey anti-rabbit MINUS (catalog no. DUO92005-30RXN Sigma) PLA probes from DuoLink were used. PLA analysis was performed using DuoLink *in situ* detection reagents Red (catalog no. DUO92008 Sigma) following the manufacturer’s protocol. Actin was stained with Alexa Fluor 488 phalloidin. Cells stained with individual primary antibodies only (no secondary antibodies) were used as negative control. None of these had red fluorescence. Coverslips were mounted using slide mounting medium and examined using a confocal microscope (LSM880; Carl Zeiss Microscopy). Fluorescence images were collected with a 63× objective lens. Quantification of PLA foci was performed using Fiji/ImageJ.

### Subcellular fractionation

Cells were grown in 60 mm dishes in serum-free medium and were incubated with 40 μM CGS9343B or an equal volume of DMSO (vehicle). After 16 h, DMSO (vehicle) or 100 ng/ml EGF was added for 5 min. Subcellular fractions were prepared using subcellular protein fractionation kit for cultured cells from Thermo Scientific (78840) according to the manufacturer’s instructions. Briefly, the cells were harvested by scraping, washed with PBS, resuspended in cytoplasmic extraction buffer, and incubated on ice for 10 min. The lysates were subjected to centrifugation at 500*g* for 5 min at 4 °C and the supernatant was collected as the cytoplasmic fraction. The cell pellet was resuspended in membrane extraction buffer, then incubated on ice for 10 min. Samples were spun at 3000*g* for 5 min at 4 °C and the supernatant was collected as the membrane fraction. Whole-cell lysates were prepared by resuspending the cell pellet in lysis buffer B, sonicated, and cleared by centrifugation at 15,000*g* for 5 min at 4 °C. The fractions were resolved by SDS-PAGE and western blotting. Blots were probed with anti-pERK, anti-ERK, anti-β-tubulin, and anti-Na^+^K^+^ ATPase primary antibodies, followed by the appropriate IRDye conjugated secondary antibodies and scanned using the Odyssey imaging system.

### Statistical analysis

All statistical analysis was performed using one-way ANOVA test using Prism 7 (GraphPad). All analyses were corrected with the Tukey’s post-hoc test. Western blot images were quantified with Image Studio 2.0 (LI-COR Biosciences) according to the manufacturer's instructions. After quantifying the bands, we calculated the ratio of each pERK band to the total ERK band in the same sample. Protein concentrations were quantified with Protein Assay Dye Reagent Concentrate (5000006, Bio-Rad).

## Data availability

All data are contained in the article.

## Conflict of interest

The authors declare that they have no conflicts of interest with the contents of this article.
